# Sex-specific effects of cytotoxic chemotherapy agents cyclophospha-mide and mitomycin C on gene expression, oxidative DNA damage, and epigenetic alterations in the prefrontal cortex and hippocampus – an aging connection

**DOI:** 10.18632/aging.100920

**Published:** 2016-03-30

**Authors:** Anna Kovalchuk, Rocio Rodriguez-Juarez, Yaroslav Ilnytskyy, Boseon Byeon, Svitlana Shpyleva, Stepan Melnyk, Igor Pogribny, Bryan Kolb, Olga Kovalchuk

**Affiliations:** ^1^ Department of Neuroscience, University of Lethbridge, Lethbridge, AB, T1K3M4, Canada; ^2^ Department of Biological Sciences, University of Lethbridge, Lethbridge, AB, T1K3M4, Canada; ^3^ Division of Biochemical Toxicology, Food and Drug Administration National Center for Toxicological Research, Jefferson, AR 72079, USA; ^4^ Department of Pediatrics, University of Arkansas for Medical Sciences, Little Rock, AR 72202, USA; ^5^ Alberta Epigenetics Network, Calgary, AB, T2L 2A6, Canada; ^6^ Canadian Institute for Advanced Research, Toronto, ON, M5G 1Z8, Canada

**Keywords:** chemotherapy, chemo brain, epigenetics, DNA methylation, DNA hydroxymethylation, oxidative stress, transcriptome, aging

## Abstract

Recent research shows that chemotherapy agents can be more toxic to healthy brain cells than to the target cancer cells. They cause a range of side effects, including memory loss and cognitive dysfunction that can persist long after the completion of treatment. This condition is known as chemo brain. The molecular and cellular mechanisms of chemo brain remain obscure. Here, we analyzed the effects of two cytotoxic chemotherapy drugs—cyclophosphamide (CPP) and mitomycin C (MMC) - on transcriptomic and epigenetic changes in the murine prefrontal cortex (PFC) and hippocampal regions. We for the first time showed that CPP and MMC treatments led to profound sex- and brain region-specific alterations in gene expression profiles. Gene expression changes were most prominent in the PFC tissues of female mice 3 weeks after MMC treatment, and the gene expression response was much greater for MCC than CPP exposure. MMC exposure resulted in oxidative DNA damage, evidenced by accumulation of 8-oxo-2′-deoxyguanosine (8-oxodG) and a decrease in the level of 8-oxodG repair protein OGG1 in the PFC of female animals 3 weeks after treatment. MMC treatment decreased global DNA methylation and increased DNA hydroxymethylation in the PFC tissues of female mice. The majority of the changes induced by chemotherapy in the PFC tissues of female mice resembled those that occur during the brain's aging processes. Therefore, our study suggests a link between chemotherapy-induced chemo brain and brain aging, and provides an important roadmap for future analysis.

## INTRODUCTION

The recent report by the American Cancer Society showed that 14.1 million new cancer cases were diagnosed worldwide in 2012, and 8 million of those occurred in developed countries. It is projected that by 2030 newly diagnosed cancer cases will reach 21.7 million worldwide (http://www.cancer.org/research/acsresearchupdates/more/10-must-know-2015-global-cancer-facts.

The development of new chemotherapeutic agents and regimens for cancer therapy has led to increasing rates of survival in cancer patients and, therefore, it is important to ensure that cancer survivors suffer minimal side effects and have good quality of life.

Despite the undisputed benefits, chemotherapy causes a wide array of side effects, among them central nervous system (CNS) toxicity [1, 2]. Recent research showed that chemotherapy agents were more toxic to healthy brain cells than to the cancer cells. Numerous studies have provided evidence of the occurrence of chemotherapy-cognitive dysfunction [3, 4]. For example, Wefel and Schagen (2012), reported that with regard to breast cancer alone, more than 60 studies found various degrees of association between chemotherapy and cognitive impairments [5].

Chemotherapy-induced CNS side effects impact the cognitive domains of attention, memory, processing speed, and executive function [6–8], causing a condition that has been termed *chemo brain* [9]. The persistence of chemo brain manifestations range from short to long [2, 10], affecting 35% of patients for months to years after the cessation of treatments. Furthermore, data by the International Cognitive Workshop indicates that chemo brain's cognitive side effects can persist for as long as five to ten years after treatment completion [4, 9]. In order to prevent and mitigate chemo brain side effects, it is important to understand the underlying mechanisms that are affected by chemotherapy agents in the brain.

The proposed mechanisms of chemo brain include increased oxidative stress, chronic inflammation, inhibition of neuronal proliferation, differentiation and disruption of hippocampal neurogenesis, induction of apoptosis, alterations in brain blood flow, changes in metabolism, disruption of blood-brain barrier, and white matter dysfunction [6, 11–14].

Although the molecular mechanisms underlying chemo brain have been assessed in clinical studies, analyses are difficult to conduct because of large inter-patient variability, different treatment protocols, disease status, and co-morbidities [15]. Thus, much of the recent chemo brain research has employed cell lines as well as rodent models (reviewed in [6, 7]). Several model-based studies have reported that chemotherapy exposure caused oxidative stress, inhibited neuronal proliferation and differentiation, increased apoptosis, and altered levels of histone modification and chromatin remodeling, thus leading to aberrant levels of neurotrophin and neurogenic proteins in the brains of experimental animals. These molecular changes were associated with altered neurogenesis and deficits in learning and memory processes [12, 16].

The frequency and timing of chemo brain occurrence and persistence suggest that chemo brain may be epigenetic in nature and associated with aberrant gene expression profiles. The vast majority of chemo brain studies have focused on the hippocampus, due to its involvement in several cognitive processes, including spatial navigation, memory processing, storage of long-term memory, and declarative memory. Yet, almost nothing is known about effects of chemotherapy on the PFC, a key regulatory region that is involved in executive functions, such as working memory, decision-making, planning, judgment, social behavior, as well as abstract thinking.

In the present study, we analyzed the effects of two cytotoxic chemotherapy drugs - cyclophosphamide (CPP) and mitomycin C (MMC) - on gene expression and epigenetic changes in the murine brain, focusing on the PFC and hippocampal regions. We demonstrated that CPP and MMC treatment led to substantial noticeable sex- and brain region-specific alterations in gene expression profiles, oxidative DNA damage, and changes in global levels of cytosine DNA methylation and hydroxymethylation.

## RESULTS

### Analysis of gene expression response to CPP and MMC treatment

Global gene expression profiling provides a mechanistic insight into a milieu of molecular processes and pathways associated with exposures to various genotoxic and non-genotoxic stressors, as well as with a variety of disease conditions. Our aim was to use an Illumina Bead Array platform to conduct an in-depth gene expression analysis of the PFC and hippocampal tissues of male and female mice three weeks after exposure to chemotherapy agents CPP or MMC.

**Figure 1:**
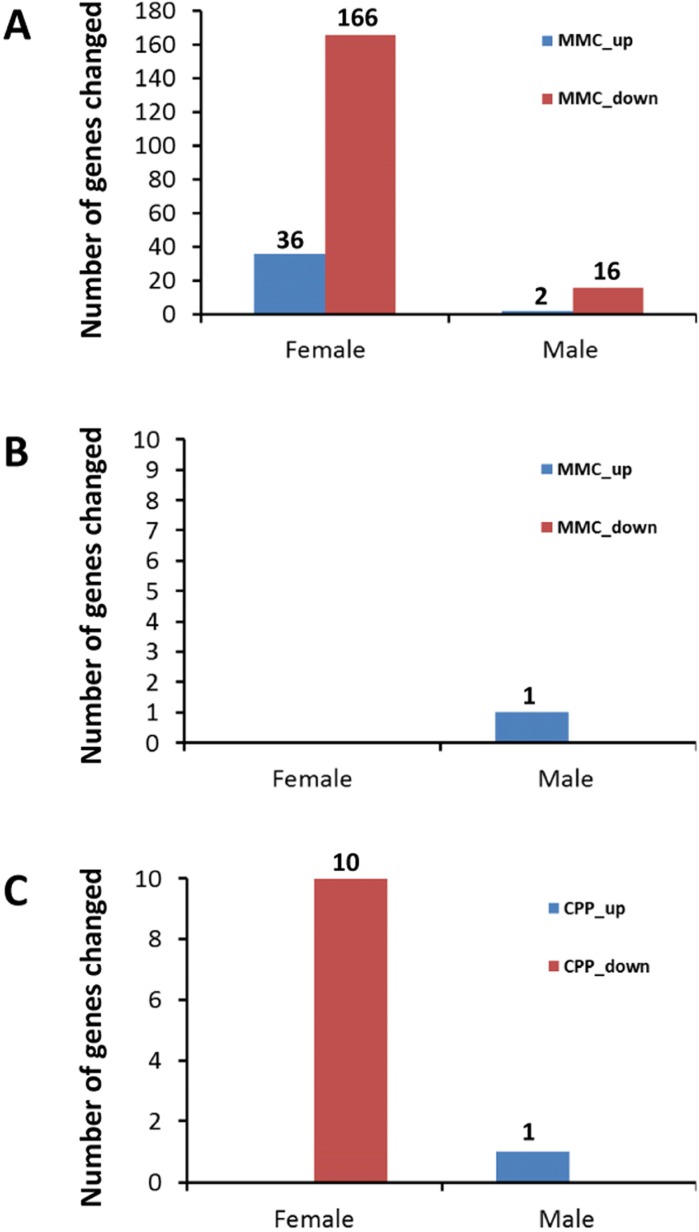
Number of up- and downregulated genes in the prefrontal cortex and hippocampus of male and female animals exposed to MMC or CPP (**A**) number of differentially expressed genes in PFC of animals in response to MMC; (**B**) number of differentially expressed genes in hippocampus of animals in response to MMC; (**C**) number of differentially expressed genes in hippocampus of animals in response to CPP.

The transcriptomic analysis revealed no notable changes in gene expression in the PFC after exposure to CPP (data not shown). In contrast, changes in gene expression in response to MMC treatment were evident three weeks after exposure (Figure 1A), especially in female mice. Thirty-six genes were upregulated and 166 genes were downregulated in female mice, while only 2 and 16 genes were upregulated and downregulated, respectively, in male mice following MMC exposure (the adjusted p-value <0.05 and fold change 1.5) (Figure 1A).

The hippocampus, in contrast to the PFC, contained almost no differentially expressed genes three weeks after MMC exposure: only a single gene, (predicted gene EG545253), was upregulated in male mice hippocampal tissues (Figure 1B). Exposure to CPP led to the upregulation of a single same gene, (EG545253), in the male mice at three weeks post treatment, while ten genes were downregulated in the hippocampal tissues of female mice (p <0.05, fold change 1.5) (Figure 1C).

Thus, the MMC-induced changes in gene expression were brain-region specific, and were seen only in the PFC and not in the hippocampus (Figures 1A 1A and 1B).

### Functional analysis of gene enrichment pathways

To further investigate the functional significance of the observed gene expression changes we conducted an in-depth pathway analysis study. First, we annotated differentially expressed genes in response to MMC or CPP treatment by performing a functional annotation Gene Ontology (GO) terms analysis using the DAVID bioinformatics platform.

Among the genes that were downregulated at three weeks post-treatment in the PFC of female mice exposed to MMC, enrichment was found in the positive regulation of the Notch signaling pathway (more than 50 fold) and in neural crest cell differentiation (more than 10 fold). In the male mice, the endoplasmic reticulum and endosome were enriched. The findings for male and female mice showed no overlap. In the upregulated genes at three weeks post treatment in the PFC tissues of female mice exposed to MMC, enrichment was observed in the GO terms of olfactory receptor activity (S. Table 1). Only a few genes were changed in the male mice to warrant the same analysis that was conducted for the female groups.

We further analysed gene expression data based on pathway knowledge the genes and networks altered in response to MMC and CPP using WikiPathways and KEGG pathways. The PFC of females exposed to MMC at three weeks showed downregulated genes belonging to dopaminergic neurogenesis (Figure S. 1) and oxidative phosphorylation pathway (Figure S. 2).

**Figure 2:**
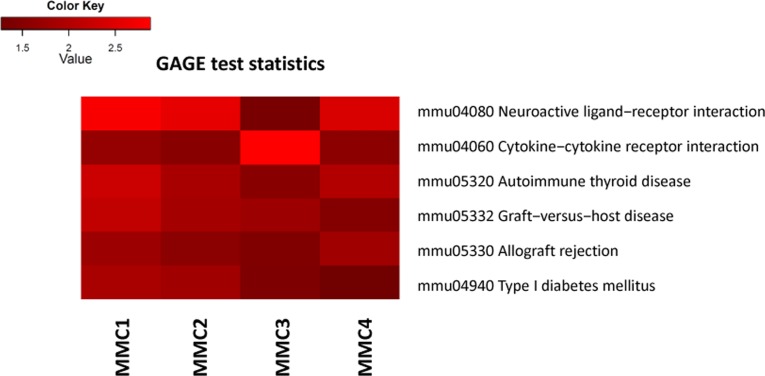
Bi-directional Generally Applicable Gene-set Enrichment (GAGE) analysis MMC 1-4 denote individual MMC-exposed animals.

Additionally, we used a Generally Applicable Gene-set Enrichment (GAGE) analysis implemented as Bioconductor package (version 2.18.0) to detect significantly perturbed metabolic and signaling canonical pathways [17]. Bi-directional analysis yielded several significantly altered pathways, such as neuroactive ligand-receptor interaction, graft-versus-host disease, allograft rejection and others (Figure 2, Figure S. 3).

### Chemotherapy-induced oxidative damage

**Figure 3:**
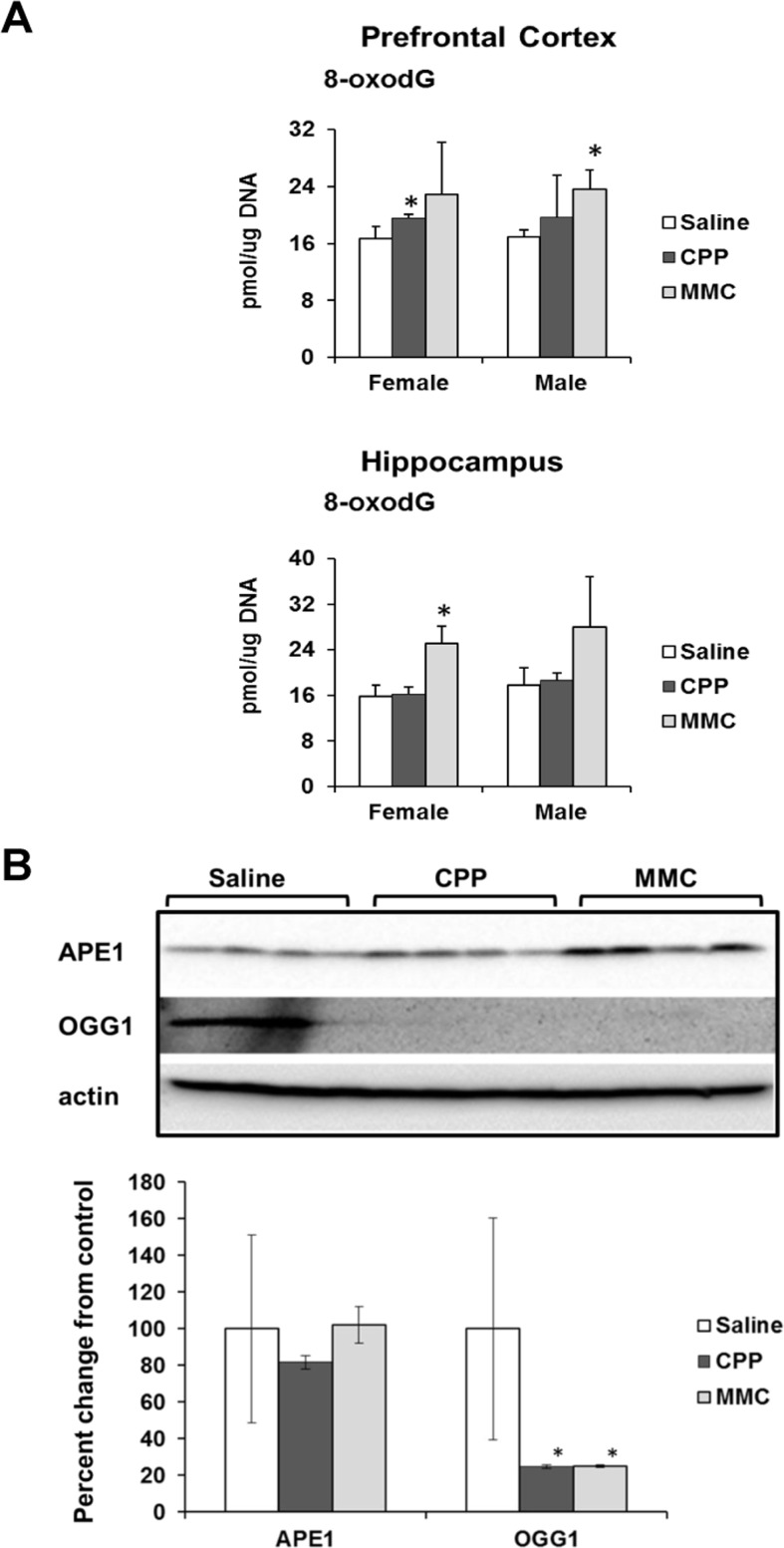
Oxidative DNA damage in the PFC and hippocampus tissues of chemotherapy-exposed animals (**A**) Levels of 8-oxo-7-hydrodeoxyguanosine (8-oxodG) in genomic DNA isolated from the PFC and hippocampus of male and female mice (mean ± SD, n=4); *p<0.05, Student's *t*-test. (**B**) Levels of APE1 and OGG1 in the PFC tissues of chemotherapy-exposed female animals 3 weeks after treatment. Lysates from PFC tissues were immunoblotted using antibodies against APE1, OGG1 and actin. *p<0.05, Student's *t*-test.

It has been reported by Kesler (2014) that the observed chemotherapy-induced gene expression changes indicate unaltered cellular redox status and oxidative stress [18]. Oxidative stress has been proposed as one of the mechanisms of chemo brain pathogenesis [19, 20]. Based on these findings, we assessed the levels of 8-oxo-2′-deoxyguanosine (8-oxodG) in genomic DNA from PFC and hippocampal tissues of MMC and CPP exposed male and female animals. The 8-oxodG molecule, one of the predominant and best-studied molecular markers of oxidative DNA damage, is formed by the action of reactive oxygen species [21]. Exposure to CPP caused a small but statistically significant (p<0.05) increase in 8-oxodG levels in the PFC tissues of female, but not male, animals at 3 weeks post-treatment. Exposure to MMC led to a statistically significant increase in the 8-oxodG levels in the PFC tissues of male mice (p=0.016), while the PFCs of MMC-exposed female mice showed a strong trend towards an increase in 8-oxodG levels, but the difference did not reach statistical significance (p=0.146) (Figure 3 A). In the hippocampus tissues, CPP exposure had no effect on the levels of 8-oxodG in either male or female animals, while MMC exposure led to significantly elevated levels in female mice (p=0.002) and an increasing, but not statistically significant trend in males (p=0.179) (Figure 3A). As such, MMC was a more potent inducer of oxidative DNA damage when compared to CCP.

We explored potential mechanisms for the persistence of 8-oxodG in the genomic DNA of the PFC tissues of the MMC-exposed animals by determining the level of key base excision repair proteins involved in the repair of oxidative DNA damage. The level of base excision repair proteins is another well-accepted marker of oxidative DNA damage [22, 23]. Our analysis of the levels of 8-oxoguanine glycosylase (OGG 1) and apurinic/apyrimidinic endonuclease 1 (APE1) using Western immunoblotting revealed a statistically significant reduction in the levels of OGG1 (p=0.045 and p=0.044) in the PFC tissues of the MMC- and CPP-exposed female animals, respectively (Figure 3B). No statistically significant changes were observed in the levels of APE1 (Figure 3B).

### Analysis of global DNA methylation in the PFC and hippocampal tissues of chemotherapy-exposed mice

Several studies have indicated that aberrant cytosine DNA methylation may occur because of oxidative DNA damage [22, 24]. Aberrant DNA methylation is also associated with altered gene expression patterns [25]. Therefore, we analyzed the status of global DNA methylation in the hippocampal and PFC tissues of CPP- and MMC-exposed male and female mice. We determined the levels of 5-methyl-cytosine (5mC) and 5-hydroxymethyl-cytosine (5-hmC) in the genomic DNA of PFC and hippocampal tissues of chemotherapy-treated animals at 3 weeks post-exposure.

**Figure 4:**
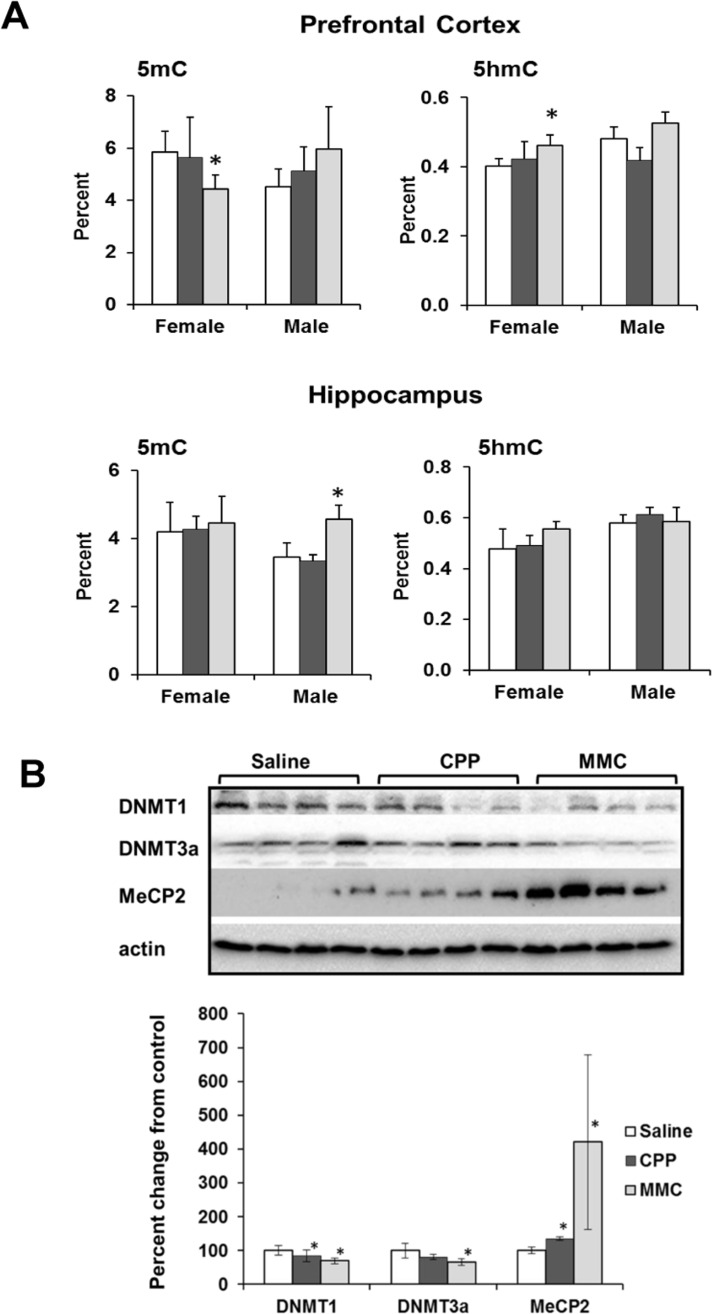
Altered DNA methylation in the PFC and hippocampus tissues of chemotherapy-exposed animals (**A**) Levels of 5-methylcytosine (5mC) and 5-hydroxymethylcytosine (5hmC) in the genomic DNA of the in the PFC and hippocampus tissues of chemotherapy-exposed animals. Mean ± SD, n = 4; *p<0.05, Student's *t*-test. (**B**) Levels of DNMT1, DNMT3a and MeCP2 in the PFC tissues of chemotherapy-exposed female animals 3 weeks after treatment. Lysates from PFC tissues were immunoblotted using antibodies against DNMT1, DNMT3a, MeCP2 and actin. *p<0.05, Student's *t*-test.

We found a statistically significant decrease in the level of 5-mC in the global DNA of PFC tissues of MMC-treated female mice (p=0.025), whereas the level of 5-hmC was significantly increased (p=0.017). MMC-treated male mice showed an increase in the 5hmC levels in the hippocampus (p=0.018), but no changes were observed in the 5mC levels. CPP treatment had no effect on the levels of 5mC or 5-hmC in any of the studied tissues (Figure 4A).

We further explored the decrease in the level of 5-mC and the increase in the level of 5-hmC in the PFC tissues of MMC-exposed females by determining the levels of proteins that establish and maintain these epigenetic modifications [26]. MMC exposure led to a significant downregulation of the cellular levels of maintenance DNA methyltransferase DNMT1 (p=0.032), and *de novo* DNA methyltransferase DNMT3a (p=0.017) in the female PFC tissues at 3 weeks post-treatment (Figure 4B). In parallel, the levels of the methyl-CpG binding protein MECP2 were increased (p=0.044). The levels of Tet1 and Tet 2 were unaffected (data not shown).

## DISCUSSION

This is the first in-depth analysis study of gene expression profiles in PFC and hippocampus tissues of male and female mice 3 weeks after treatment with the chemotherapy agents MMC and CPP. The key findings of our study are: (i) chemotherapy altered the gene expression profiles in the murine PFC and hippocampus tissues; (ii) gene expression changes were most prominent in the PFC tissues of female animals 3 weeks after MMC treatment; (iii) the magnitude of gene expression response was much greater for MCC than CPP treatment; (iv) MMC treatment resulted in accumulation of 8-oxodG, decreased global DNA methylation, and increased DNA hydroxymethylation in the PFC tissues of female animals; and (v) the majority of the changes induced by MMC in the PFC tissues of female mice resembled those that occur during the aging processes. As such, the PFC of both male and female animals appears not to be sensitive to CPP treatment, although the reasons for this apparent resistance require further analysis.

Our data suggest that the PFC of females may be more vulnerable in the long term, as the significant changes observed in females at 3 weeks post-exposure to MMC were not apparent in males. We showed that MMC exposure leads to profound alterations in the global gene expression, affecting pathways responsible for oxidative stress and others. We also demonstrated that MMC exposure leads to accumulation and persistence of 8-oxodG in the PFC tissues of female animals at 3 weeks after exposure. This accumulation was paralleled by decreases in levels of 5-methylcytosine and increases in levels of 5-hydroxymethylcytosine.

The elevated level of the oxidative stress biomarker 8-oxodG and the decreased level of key BER protein (OGG1) that repairs 8-oxodG strongly infer that MMC induces oxidative DNA damage and oxidative stress [22, 27]. The 8-oxodG is a prevalent and well-studied component of oxidative DNA lesions [21]. Various studies have established the important role of altered cellular redox status in the pathophysiology of major neurological diseases and conditions, such as Alzheimer's disease, Parkinson's disease, stroke, autism, and many others [22, 28–30]. Oxidative stress is also viewed as one of the potential mechanisms of chemo brain [19, 20], but the associated processes and pathways linking oxidative stress and chemo brain have not been analyzed beyond claiming an effect of chemotherapy treatment on the brain.

The present study, for the first time, shows a persistent decrease in the levels of OGG1 in the PFC of chemotherapy-exposed female animals. OGG1 is a glycosylase involved in the initial steps of recognition and removal of 8-oxodG, a cytotoxic and mutagenic lesion, by the highly conserved base excision repair pathway [31]. OGG1 is the first enzyme in the short-patch BER; thus, the success of 8-oxodG removal heavily depends on proper OGG1 function. *OGG1* is highly abundant in the brain, where it functions to protect neurons against oxidative DNA damage and apoptosis and to maintain proper neuronal connectivity [28]. OGG1 is very important for brain development. As well, the loss or inhibition of OGG1 and subsequent accumulation of 8-oxodG in the genome are associated with cancer, neurodegenerative diseases, metabolic diseases, obesity, and autism [22, 29, 31, 32]. Along with these and other pathological conditions, OGG1 loss has also been implicated in brain aging [28, 33].

Another key finding of the present study is the decrease in 5-mC and concurrent increase in 5-hmC in the PFC tissues of female mice 3 weeks after exposure to MMC. Ours is the first study to report changes in these epigenetic markers in context of chemo brain. The observed decrease in 5mC, indicative of global DNA hypomethylation, may be attributed to the observed reduction in the levels of maintenance and *de novo* DNA methyltransferases DNMT1 and 3a. It also may reflect the presence of oxidative lesions themselves that were previously demonstrated to decrease the expression and activity of DNMTs, leading aberrant DNA methylation patterns.

The reduced levels of DNMT1 and DNMT3a constitute anther important finding. These proteins are important for maintenance of DNA methylation and synaptic plasticity in the adult brain [34]. Their reduced expression has been associated with blast-induced neurotrauma, loss of synaptic functions in forebrain neurons[35], and several neurodegenerative diseases and brain aging. Inactivating mutations in DNMT1 are associated with hereditary sensory neuropathy and adult-onset dementia [36].

The observed persistent increase in the levels of 5hmC is also important, because changes in DNA hydro-xymethylation have been implicated in neuro-degeneration and brain aging [37, 38]. Recent studies link decreases in 5mC and increases in 5-hmC to newly discovered demethylation functions of TET proteins, and TET-mediated enzymatic oxidation of 5mC [25, 39]. Nevertheless, the cellular levels of Tet proteins were unchanged following MMC exposure in the present study. Two recent investigations reported similar accumulations of 5hmC in brain tissues without changes in the TET levels [22, 40]. Thus, the increase in 5hmC observed in the present study is perhaps not associated with its role in DNA demethylation. Its precise role, as well as its locus specificity, need further clarification.

**Figure 5:**
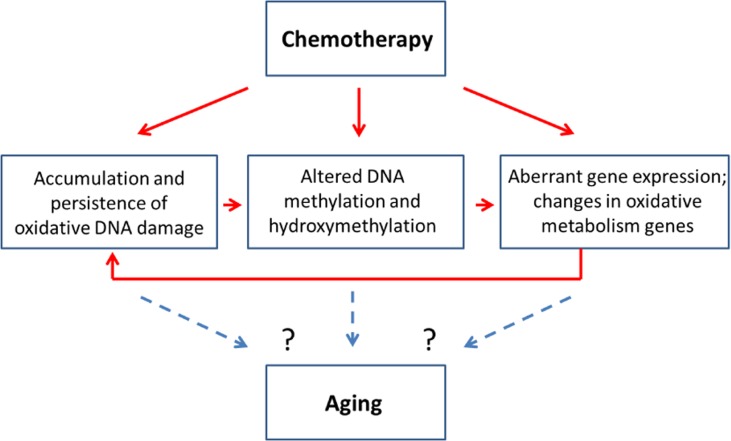
Chemotherapy-induced changes may be connected to the aging-related changes - model scheme

Altered cellular levels of 5mC and 5 hmC, in turn, may affect gene expression as well as genome stability, emphasizing the importance of analyzing the precise locus-specific distribution and plasticity of these changes in chemo brain and other neurological conditions. Increased oxidative stress, altered methylation and aberrant gene expression were all observed in the PFC of MMC-exposed female animals 3 weeks post-treatment. This may indicate possible interconnectivity of these molecular processes. Indeed, MMC can cause oxidative damage, which leads to altered DNA methylation and aberrant gene expression. The aberrant expression of oxidative pathways, as shown in our study, may also lead to accumulation of oxidative damage, thereby creating a ‘damage loop’ and causing persistence of molecular changes in the PFC (Figure 5).

The cytotoxic chemotherapy-induced changes observed in the PFC constitute yet another seminal finding. The vast majority of previous chemo brain studies focused on the hippocampus, due to its role in neurogenesis, whereas the PFC, a key regulatory region, has been under-investigated. Several clinical research articles pointed out an important role of the PFC in chemo brain [41, 42], and one mouse model-based analysis reported PFC effects upon application of a targeted cancer drug everolimus [43]. The current study opens new avenues for the analysis of chemo brain and the role of PFC damage in its etiology and pathogenesis.

The molecular changes observed in this chemo brain study have also been reported to play key parts in neurodegeneration and aging (Figure 5). Indeed, increased oxidative damage and altered levels of DNA methylation and hydroxymethylation are established signs of aging [33, 38, 44]. Furthermore, a recent clinical research article suggested a link between aging and cancer treatments and called for basic and model-based research to dissect the link between these two key clinical conditions [18]. In light of this need, our study establishes the initial mechanistic links between chemotherapy-induced chemo brain and brain aging, and provides an important roadmap for future analysis.

## MATERIALS AND METHODS

### Animals and treatment

Forty-five days old BALB/c male and female mice, average weight 25-27 grams and 22-24 grams, respectively, were housed in a temperature-controlled (24°C) room with a 12-h light-dark cycle. The mice were randomly allocated to the following groups: (i) MMC-treated; (ii) CPP-treated; (iii) controls (10 males and 10 females). CPP and MMC doses were calculated using the body surface area conversion from approximating the recommended human clinical dose levels in the range of 400-1800 mg/m^2^ (http://reference.medscape.com/drug/cytoxan-cyclophosphamide-342214) and 20 mg/m2 (http://reference.medscape.com/drug/mitomycin-342124), accordingly.

Animals from treated groups received intraperitoneal injections of either MMC (Sigma-Aldrich, St. Louis, MO; 5 mg/kg) or CPP (Sigma-Aldrich; 200 mg/kg) as 2 consecutive injections every other day, and were euthanized 3 weeks after chemotherapy to examine the persistent effects on the brain. The brains were excised and dissected, and the PFC and hippocampal tissues were immediately frozen in liquid nitrogen and stored at −80°C for subsequent analyses. All animal experimental procedures were approved by the University of Lethbridge Animal Welfare Committee.

### Gene expression analysis

The hippocampus and PFC tissues of four animals per group were used for the analysis of gene expression profiles. Differential expression analyses were performed by the Illumina® GenomeStudio software using an Illumina-custom model. In brief, RNA was extracted from the hippocampus and the PFC tissues using TRIzol® Reagent (Invitrogen, Carlsbad, CA), further purified using an RNAesy kit (Qiagen), and quantified using Nanodrop2000c (ThermoScientific). Afterwards, RNA integrity and concentration were established using 2100 BioAnalyzer (Agilent). Gene expression profiles were determined using Illumina MouseRef-8 v2.0 Expression BeadChip. Differential expression analyses were performed by the Illumina® GenomeStudio software using an Illumina-custom model. To process the data, all expression values were made positive by adding an offset (minus minimum value plus one). Next, all expression values were transformed into Log2 values and normalized by the quantile method. To reduce the number of differentially expressed genes and to increase the statistical significance of the outcome, the genes whose detection p-values in all the 8 compared samples (4 controls and 4 treatments) were greater than or equal to 0.01 were removed. The adjusted p-values from moderated t-statistics [45] were calculated using linear model and Bayes moderation. For the identification of differentially expressed genes, we have compared treatment samples (MMS and CPP) to appropriate sex (male or female), tissue type (prefrontal cortex or hippocampus) and controls (treated with saline). Differentially expressed genes were then extracted on the basis of the adjusted p-value 0.05 and fold change 1.5.

Several comparisons within control groups were made; these included comparison between male and female control samples, as well as between hippocampus and prefrontal cortex. The functional annotations of differentially expressed genes were performed using DAVID, GO (Gene Ontology) Elite, and GO-TermFinder [46]. Pathways were visualized using WikiPathways and DAVID Bioinformatics Resources 6.7 KEGG Pathways [47].

The Generally Applicable Gene-set Enrichment (GAGE) analysis implemented as Bioconductor package (version 2.18.0) was used to detect significantly perturbed metabolic and signalling canonical pathways [17, 48]. Gene set data containing KEGG pathways was obtained using the gageData Bioconductor package version 2.6.0. In order to detect significantly altered KEGG pathways, the matrix of normalized microarray expression values was loaded into R and processed using ‘gage’ function used in GAGE Bioconductor package with the following options specified: ‘kegg.sets.mm’ as a set of canonical pathways, saline treated samples used as reference, set size 10 to 500, experimental design was specified as “unpaired”, fold changes were used as per gene statistics. Gage function was applied twice: once to detect pathways changed in the same direction (same.dir=T) and, alternatively, to detect pathway with bi-directional changes, where part of the genes are up- and downregulated with no clear direction (same.dir=F). Significantly perturbed KEGG pathways were drawn using pathView Bioconductor package version 1.8.0 [48].

### Analysis of 8-oxo-7-hydrodeoxyguanosine, 5-methylcytosine, and 5-hydroxymethylcytosine in cerebellar DNA

DNA was extracted from PFC and CPP tissues using the Qiagen DNeasy Kit. The levels of 8-oxodG, 5mC, and 5hmC in mouse PFC and hippocampal tissue DNA were measured by liquid chromatography combined with electrospray tandem mass spectrometry (LC-MS/MS) as described previously [49].

### Western immunoblotting

Western immunoblotting was conducted as described previously [50]. The membranes were incubated with primary antibodies against APE1, OGG1, DNMT1, DNMT3a, MeCP2 (1:1000, Abcam) and actin (1:2000, Abcam) overnight at 4° C. Primary antibody binding was detected using horseradish peroxidase-conjugated secondary antibodies and the Enhanced Chemiluminescence Plus System (Amersham Biosciences, Baie d'Urfé, Quebec). Chemiluminescence was detected using a FluorChem HD2 camera with FluorChem software (Cell Biosciences). The membranes were stained with Coomassie blue (BioRad, Hercules, CA) to confirm equal protein loading. Signals were quantified using NIH Image J64 software and normalised relative to actin or Coomassie staining.

## SUPPLEMENTARY DATA


