# Supplementary material for: Sex-specific effects of cytotoxic chemotherapy agents cyclophospha-mide and mitomycin C on gene expression, oxidative DNA damage, and epigenetic alterations in the prefrontal cortex and hippocampus – an aging connection
Source: Aging (Albany NY). 2016 Mar 30;8(4):697–708. doi: 10.18632/aging.100920 (PMC4925823; doi:10.18632/aging.100920)
Supplement: Supplementary file 1 [file aging-08-697-s001.pdf]

SUPPLEMENTARY DATA

Table 1. DAVID annotation analysis.

Term	Count	%	P-value	Fold Enrichment
**PFC_MMC_Female_ 3w_Up**				
olfactory receptor activity	5	16.67	0.037	3.56
**PFC_MMC_Female_3w_Down**				
positive regulation of Notch signaling pathway	2	1.45	0.036	54.86
neural crest cell differentiation	3	1.45	0.028	11.35
**PFC_MMC_Male_3w_Down**				
endoplasmic reticulum part	3	21.43	0.006	20.63
Endosome	3	21.43	0.009	17.40
**HIPP_CPP_Female_3w_Down**				
zinc-finger	4	40	0.013	6.62
zinc ion binding	4	40	0.049	3.77
von Willebrand factor, type C	3	30	1.83E-04	130.26

"Count" - shows the number of genes belonging to a defined GO:term in a particular comparison group, "%" – shows the percentage these genes represent out of all genes changed in a particular comparison group, "P-value" – shows significance (only the GO:terms that had p-value lower than 0.05 are shown) and "fold enrichment" – shows fold enrichment (FE) of a certain pathway, calculated according to the following formula: FE = n/N / x/X, where n – the number of genes involved in a given pathway in a given sample, N – the total number of genes changed in a given sample, x – the total number of genes in the genome belonging to a given pathway, X – the total number of genes in the genome.

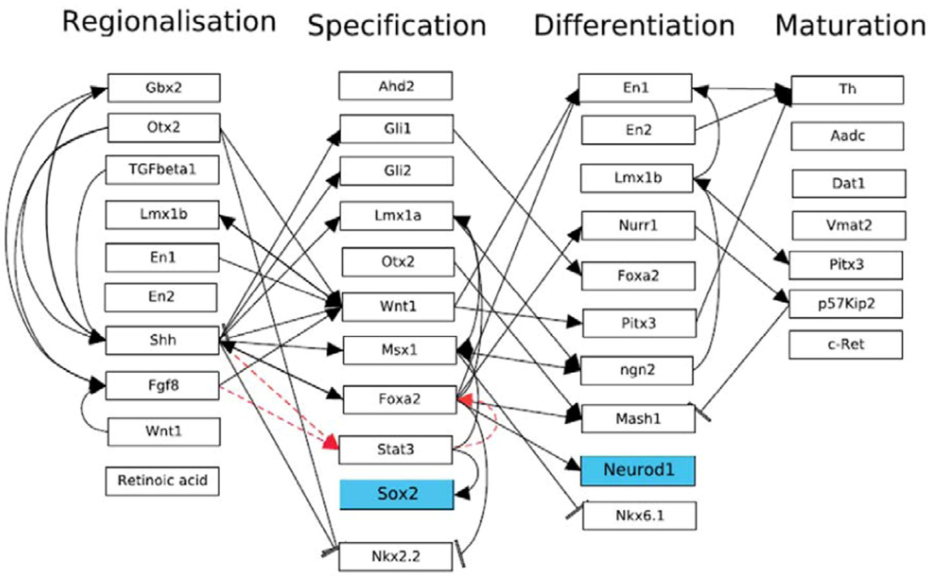

Figure S1. Visualization of genes downregulated in Dopaminergic Neurogenesis pathway in the PFC of females 3 weeks after MMC exposure (WikiPathways). Downregulated genes are shown in blue. MMC1-4 refers to individual animals subjected to MMC treatment.

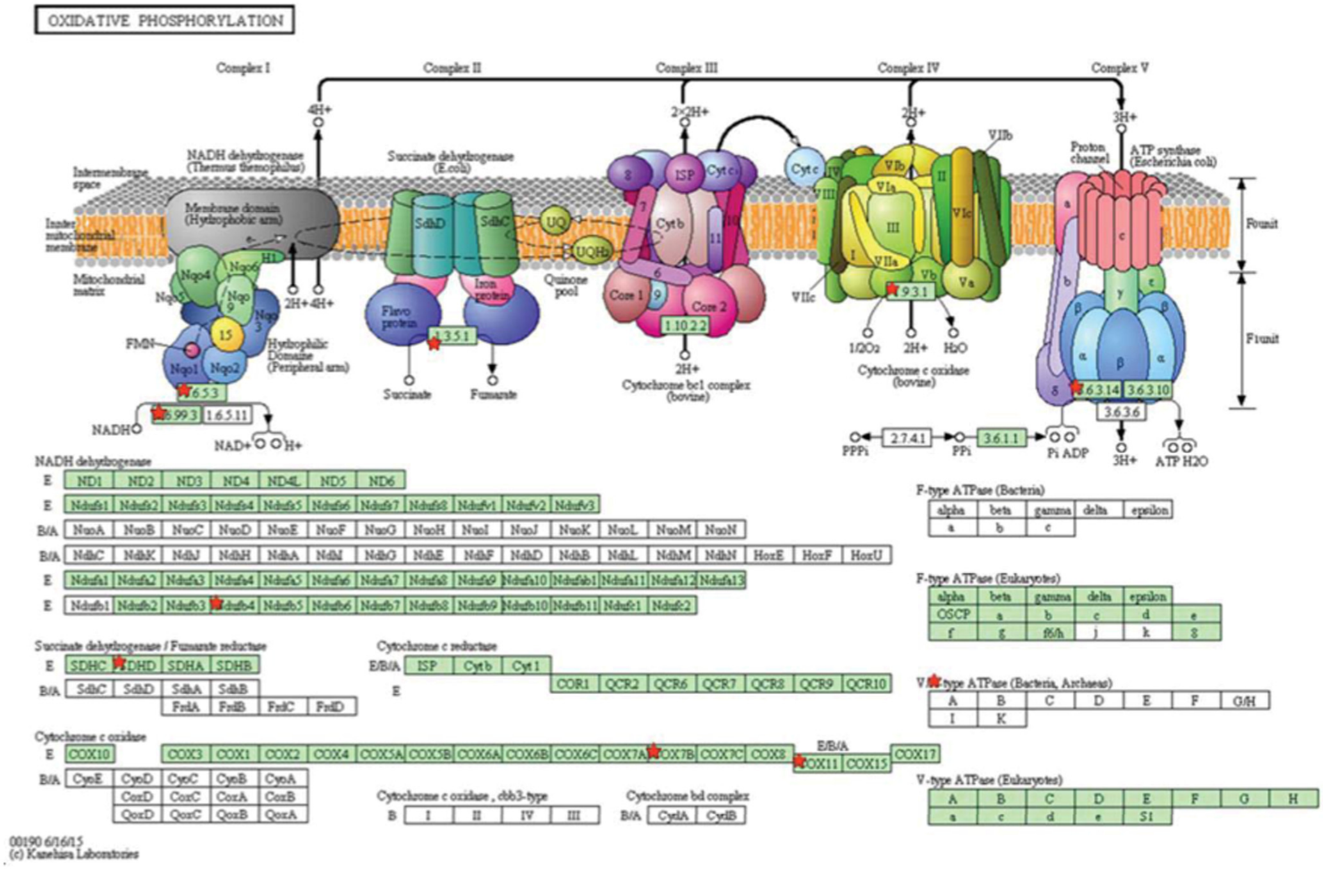

Figure S2.Visualization of genes downregulated in the KEGG oxidative phosphorylation pathway in the PFC of females 3 weeks after MMC exposure (DAVID Bioinformatics Resources 6.7)[19, 50]. Stars denote genes that were downregulated in the PFC tissues of MMC-exposed female animals.

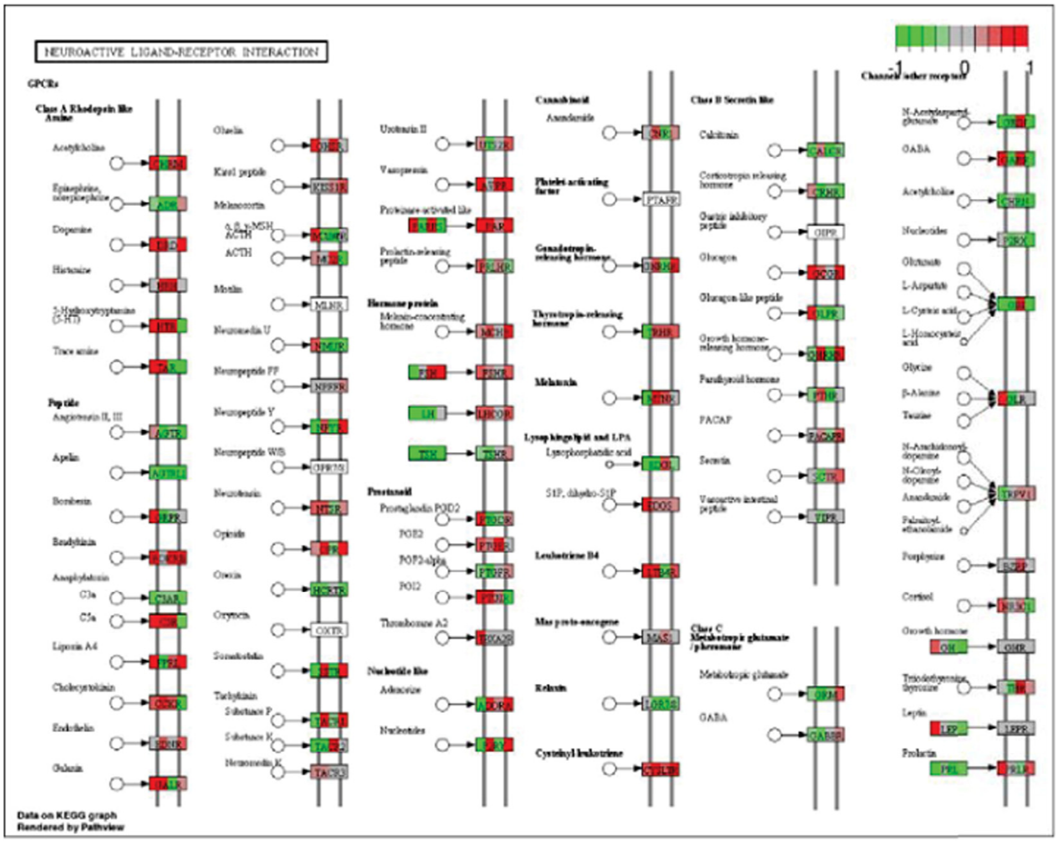

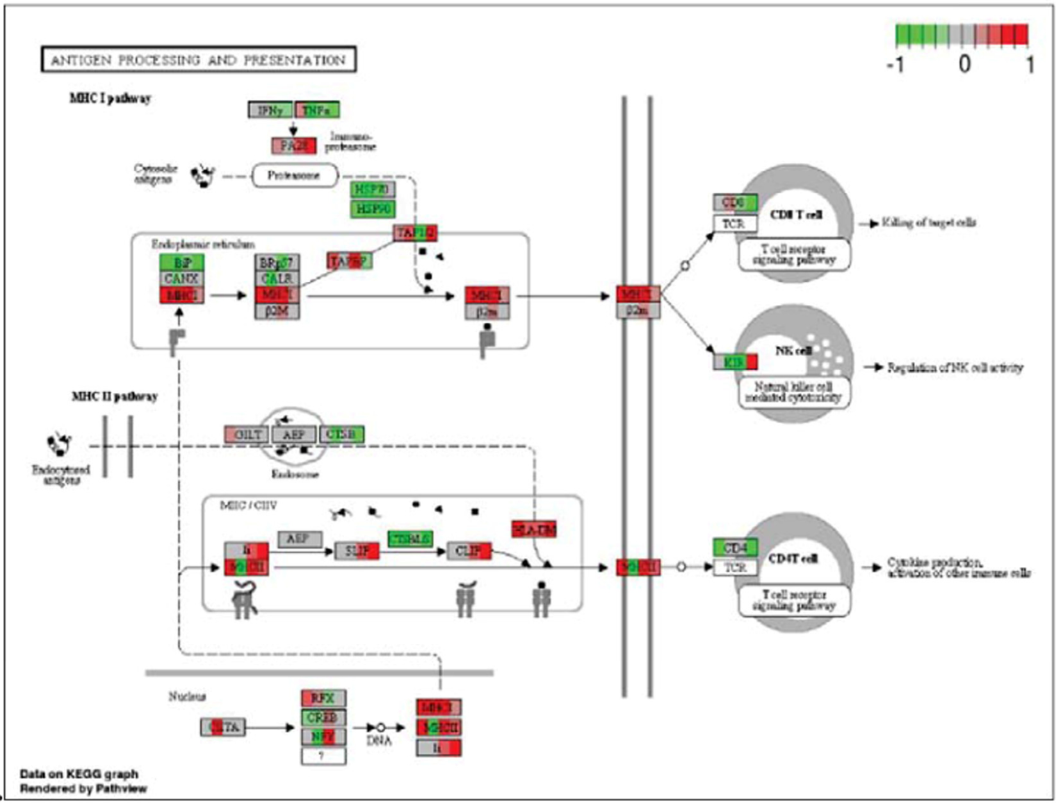

Figure S3. Bi-directionally perturbed pathways.
